# Special Issue “Fabrication and Characterization of Nanostructured Carbon Electrodes”

**DOI:** 10.3390/nano13212849

**Published:** 2023-10-27

**Authors:** Meltem Yanilmaz, Jiadeng Zhu

**Affiliations:** 1 Textile Engineering, Istanbul Technical University, Istanbul 34217, Turkey; 2 Smart Devices, Brewer Science Inc., Springfield, MO 65806, USA; jzhu14@ncsu.edu

The nanostructured carbon materials are promising electrode materials and have been widely studied owing to their tailorable structures, which offer large active sites and reduce the path of transport for mass and charge and thus provide fast pathways for electrons in rechargeable batteries and supercapacitors [1,2]. Engineering nanostructured carbons with porous structures could further increase surface area, enhance electron transfer, and thus improve electrochemical performance. Additionally, designing nanocomposite structures with various active materials and techniques has been proven to effectively fabricate high-performance electrodes [3,4,5]. This Special Issue aims to provide original contributions on synthesizing and applying nanostructured electrodes for energy storage systems.

Our Special Issue covers nanostructured electrodes, including carbon nanofibers, graphene, bio-derived carbons, and carbon composite materials. It also includes the performance of these materials in lithium-ion batteries, sodium-ion batteries, and supercapacitors. This Special Issue provides five outstanding papers that describe the development of nanostructured carbon electrodes and present their performance.

Kim et al. [6] synthesized flexible carbon nanofiber-based electrodes with Ge and MoS_2_ nanoparticles incorporated into this novel design, which led to excellent cycling performance in 200 cycles with a high capacity of over 400 mAh g^−1^ in sodium-ion batteries. In addition, the fibrous structure facilitates the fast transfer of ions and electrons and ensures that the material exhibits excellent cyclic stability and rate performance. Flexible carbon nanofibers with fine fiber diameters of approximately 200 nm accommodate the volume expansion of Ge nanoparticles and provide more active sites, thereby improving the specific capacity and cyclic stability of binder-free electrode materials. MoS_2_ further improved the reversible capacity and C-rate performance owing to its high theoretical capacity and nano-sized anchoring on the flexible CNFs.

Abdolrazzaghian et al. [7] introduced the novel design by combining centrifugal spinning and hydrothermal synthesis to fabricate nanostructured electrodes for lithium-ion and sodium-ion batteries. Highly porous graphene-containing carbon nanofibers were synthesized, and MoS_2_ was anchored on these fibrous electrodes. MoS_2_-decorated graphene-containing porous CNFs improved the electrochemical capacity up to 860 mAh g^−1^ in Li-ion cells and 455 mAh g^−1^ in Na-ion cells with excellent cycling performance. It has been proven that combining centrifugal spinning and hydrothermal synthesis techniques is a promising way to fabricate high-performance electrode materials. Zhang et al. [8] used a particular type of biomass precursor to synthesize an activated carbon featuring a three-dimensional hollow fiber comprising mesopores and micropores, exhibiting the highest specific surface area of 3635 m^2^ g^−1^. The hierarchical porous structure provides effective and sufficient spaces for tailoring pore-ion size compatibility and the use of ionic liquid electrolytes, and thus fast ion transport and diffusion were delivered, leading to a high specific capacitance of 423 F g^−1^ at 5 mA cm^−2^ in 6 M KOH electrolyte and a superior rate performance for a small-ion-size electrolyte (DMPBF4) in supercapacitors. Arena et al. [9] synthesized electrochemically active conducting inks by using commercially available polypyrrole and graphene nanoplatelets blended with dodecylbenzenesulfonic acid. The resultant electrodes presented both pseudocapacitive and electric double layer behavior, with areal capacitance of up to ~250 mF·cm^−2^ It has been noted that along with the possibility of further improvement in terms of the capacitance by adjusting the concentrations and by optimizing the technology, the reported technique is promising in view of the application of the developed inks in solid-state flexible supercapacitors. Tin (II) sulfide (SnS_2_) has attracted remarkable attention for high-energy lithium-ion batteries due to its enormous resources and simplicity of synthesis, in addition to its high theoretical capacity. However, SnS_2_ has poor intrinsic conductivity, a large volume transition, and a low initial Coulombic efficiency, resulting in a short lifespan. SnS_2_/carbon composites have been considered to be the most promising approach to addressing the abovementioned issues. Mahmud et al. [10] have summarized the current progress in the synthesis of SnS_2_/carbon anode materials and their Li-ion storage properties. They focused on the morphological structure of the SnS_2_ material. In the case of pure SnS_2_-based anodes, particle size has a significant impact on discharging capacity. SnS_2_, with a smaller particle size, showed better capacity retention and discharging capacity, and also provided a more specific area for volume change expansion. Nanoflower-based structures with more active sites for Li-ion insertion significantly improved capacity retention and discharging capability at high current rates. In the case of hybrid materials, the specific area and morphology of the other components also play a vital role in capacity performance. This Special Issue addresses a critical topic in designing high-performance electrodes and provides novel approaches that can become a reference for future studies.
